# Outcome and prognostic factors of pediatric patients with Hodgkin lymphoma: a single-center experience

**DOI:** 10.1186/s43046-023-00189-w

**Published:** 2023-09-11

**Authors:** Nesreen Ali, Mohamed Mansour, Ehab Khalil, Emad Ebeid

**Affiliations:** 1 Department of Pediatric Oncology and Hematology, National Cancer Institute, Cairo University, Cairo, Egypt; 2 Department of Pediatric Oncology and Hematology, Children Cancer Hospital Egypt (CCHE —57357), Cairo, Egypt; 3 Department of Radiation Oncology, National Cancer Institute, Cairo University, Cairo, Egypt

**Keywords:** Hodgkin lymphoma, Pediatric, Outcome, Prognostic factors

## Abstract

**Background:**

Hodgkin lymphoma (HL) is a highly curable malignant tumor. Risk-adapted treatment for children with HL aims to maximize survival while minimizing toxicity. The purpose of this study is to evaluate the outcome and prognostic characteristics of Egyptian pediatric HL patients treated at the National Cancer Institute (NCI), Cairo University.

**Methods:**

All newly diagnosed cases of classic HL treated between January 2016 and December 2018 were included in this study.

**Results:**

The median age at initial presentation was 8 years in 69 eligible individuals, with a male-to-female ratio of 4.7:1. Eighteen percent of patients had an elevated erythrocyte sedimentation rate (ESR) of more than 50, 42% had more than three lymph node (LN) group involvements, 18.8% had bulky disease, 52.2% were at an advanced stage, and 34% had B symptoms. Age  > 15 years, B symptoms,  > 3 LN group involvement, extra-nodal disease, and advanced stages significantly affected the overall survival rate (OS) (*P*-values = 0.03, 0.033, 0.008, 0.017, and 0.032). There was no statistically significant difference between patients who got combined modality therapy (CMT) and those who received chemotherapy alone (3-year OS and event-free survival (EFS) were 95.5% and 87.6% vs. 89.9% and 83.3%, *P*-values of 0.70 and 0.90). Patients with an interim-negative positron emission tomography-computed tomography (PET-CT) had a 3-year OS of 94.7%, compared to 74.1% in patients with an interim-positive PET-CT (*P* = 0.06), suggesting that rapid early response (RER) is a significant prognostic factor. There was no statistically significant survival difference between patients with a negative interim PET-CT who got CMT and those who received chemotherapy alone (3-year OS and EFS: 100% and 88.2% vs. 95% and 90%; *P* = 0.35 and 0.70, respectively). Three-year OS was 93.3% and 100%, and EFS was 74.3% and 100% (*P* = 0.495 and 0.196%) for those who got 15 Gy versus those who received 20 Gy or more, respectively. At the end of the study, the OS and EFS at 3 years for the whole group were 91.9% and 83.6%.

**Conclusion:**

Treatment with risk- and response-adaptive treatment should be the standard of care for treating pediatric patients with HL.

## Introduction

HL accounts for around 40% of all pediatric lymphomas and is the most prevalent cancer among adolescents and young adults. With combinations of chemotherapy and radiation, HL is roughly 80% curable, placing it among the most treatable cancers [1]. Since the introduction of combination chemotherapy treatments 20 years ago, the prognosis for children with HL has improved. The treatment is mostly influenced by the stage of the disease at diagnosis, histology, existence of “B”-symptoms, and the presence of bulky disease. Nonetheless, 20% of patients do not achieve long-term remission, and around 20% experience treatment-related side effects such as secondary malignancies, infertility, cardiovascular disease, and organ malfunction following chemoradiation [2]. Studies of long-term therapy side effects were made possible by the significant number of survivors. The goal of therapy optimization protocols for pediatric patients with HL is to maintain excellent tumor control while limiting adverse effects and long-term consequences [3]. It is critical to stratify patients based on reliable prognostic factors at presentation and according to the rapidity of response into low-risk (LR) patients who would benefit from less aggressive therapy, avoiding unnecessary toxic side effects, and high-risk (HR) patients who should be subjected to intensified therapy to reduce the rate of treatment failures and relapses [4]. This study aims to assess the outcome of pediatric patients with HL treated with risk- and response-adjusted therapy, determine if radiotherapy could be safely omitted for early responder patients without compromising outcome, and assess the relationship between different prognostic factors and outcome.

## Patients and method

Patients diagnosed with classic HL and treated at the NCI, Cairo University, Egypt, between January 2016 and December 2018, were recruited in the study. The patients’ demographic, clinical, and histological features, B symptoms (fever, night sweats, and weight loss), extra-nodal disease, treatment options, and results were evaluated retrospectively. After receiving clearance from the institutional review board, the study was initiated. The diagnosis of HL was determined by histological analysis of biopsies taken from patients. The disease at presentation was staged in accordance with the Ann Arbor staging criteria. The staging approach included a PET-CT scan, bone marrow aspirate, and biopsy. Risk-adapted and response-based therapy allocates the intensity of treatment based on disease parameters such as stage of disease, bulkiness, the presence of B symptoms, and early response after two cycles of chemotherapy as determined by PET-CT. Patients in the study were treated based on their initial risk and response to two courses of chemotherapy. Until the end of 2016, combined modality treatment was the standard of care for all patients; after that, response-based therapy was adopted, and only patients with a slow early response (SER) received radiation therapy. On days 1 and 15, all patients received intravenous ABVD (doxorubicin 25 mg/m^2^, bleomycin 10 U/m^2^, vinblastine 6 mg/m^2^, and dacarbazine 375 mg/m^2^). If the mediastinal mass measured greater than one-third of the transverse thoracic diameter on chest-x-ray (CXR), bulky mediastinal disease was identified. If a peripheral lymph node is larger than 6 cm, it is defined to be bulky. Patients were stratified into LR, which was defined as non-bulky stage IA or IIA illness; IR, which was defined as stages IB or IIB; bulky stage IA or bulky stage IIA disease; stage IIAE; stage IIIA regardless of size; and HR disease, which was defined as stages IIIB or IV. In the risk stratification, the letter A denotes the absence of B symptoms, letter B denotes the presence of B symptoms, and letter E denotes direct extranodal extension. The number of cycles was selected based on risk stratification: four for low-risk and six for intermediate- and HR individuals. In eligible patients for involved-site radiation therapy (ISRT), radiation doses ranged from 15 to 25 Gy. After two cycles, PET-CT was done for all patients to evaluate response (interim PET-CT). RER are patients with a complete response (CR) after two cycles of chemotherapy, as evaluated by a PET scan with Deauville scores 1, 2, or 3. Progressive disease (PD) was defined as at least one initially involved mass increasing by more than 25% compared to the best previous response, or the appearance of new lymphatic or extra-lymphatic lesions, or the recurrence of  B symptoms that  cannot be explained by other factor. Refractory disease was defined as relapse occurring during or within 3 months of treatment, early relapse occurred between 3 and 12 months, whereas late relapse occurred after 12 months following treatment.

### Statistical analysis

We evaluated differences between demographic and clinical variable distribution subgroups using the 2-test or Fisher's exact test for dichotomous data. The Kaplan–Meier method was used to estimate EFS and OS, and the log-rank test was used to compare the two outcomes across groups. All provided values are two sided. All variables result in a *P*-value. OS rates will be computed from the date of diagnosis to the date of death from any cause; patients who are still alive or who have been lost to follow-up will be censored on their last known date of survival. While EFS will be measured from the date of attaining remission to the date of progression, relapse, or death, whenever occurs first, patients who did not progress, relapse, or die will be censored at the final evaluation before to loss to follow-up. Every *P*-value is two sided. *P*-values less than 0.05 will be considered significant.

## Results

Fifty-seven of the 69 investigated patients were male, while 12 were female. The median age was 8 years old (range: 3–17 years). The majority of cases were nodular sclerosis (33 cases; 47.8%), followed by mixed cellularity (29 cases; 24.1%) and lymphocyte rich (3 cases; 5.8%). Four patients (5.7%) had typical HL, but no pathological subtype was known. The frequency of stage IV was highest among investigated patients (24 cases; 34.8%), followed by stage I (21 cases; 30.4%), stage III (12 cases; 17.4%), and stage II (12 cases; 17.4%). Early stages (including stages I and II) accounted for 48% of the total cases, whereas advanced stages (containing stages III and IV) accounted for 52%. LR (27 patients; 39.1%), IR (15 patients; 21.7%), and HR (27 patients; 39.1%) were observed. Initial B symptoms were present in 34.8% of patients and missing in 65.2% of individuals. Initial bulky disease was seen in 13 individuals (18.8%) but absent in 56 (81.2%). The majority of patients (68.1%) received chemotherapy alone, whereas only 31.9% received both chemotherapy and radiation. The characteristics of the studied patients are shown in Table 1.

**Table 1:** Patient’s characteristics

Characteristics	*n*	Percentage
**All**	69	100%
**Gender**		
Male	57	82.6%
Female	12	17.4%
**Age groups**		
0–5 yrs	13	18.8%
> 5–10 yrs	29	42.1%
> 10–15 yrs	21	30.4%
> 15 yrs	6	8.7%
**ESR**		
< 50	56	81.2%
≥ 50	13	18.8%
**LN no**		
< 3	40	58.0%
≥ 3	29	42.0%
**Bulky disease**		
No	56	81.2%
Bulky peripheral	5	7.2%
Bulky mediastinal	8	11.6%
**Stage**		
I	21	30.4%
II	12	17.4%
III	12	17.4%
IV	24	34.8%
**B symptoms**		
No	45	65.2%
Yes	24	34.8%
**Risk classification**		
HR	27	39.1%
IR	15	21.8%
LR	27	39.1%

*LR* low risk, *IR* intermediate risk, *HR* high risk, *LN* lymph node

**Fig. 1:**
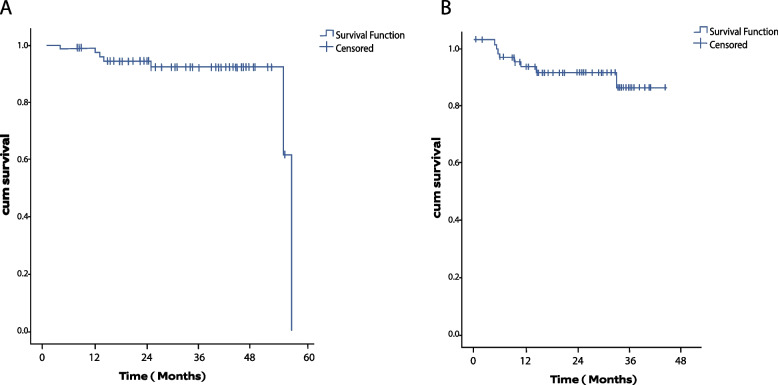
**A** Overall survival of the whole cohort. **B** Event-free survival of the whole cohort

**Fig. 2:**
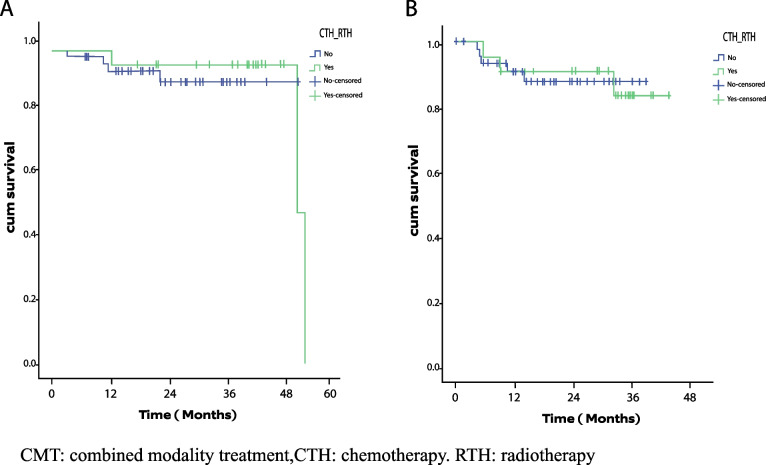
**A** Overall survival of patients who received CMT versus received chemotherapy alone. **B** Event-free survival of patients who received CMT versus received chemotherapy alone

The majority of cases (60 patients; 89%) were RER (CR with PET-CT negative after the second cycle of ABVD), while nine patients (13%) were SER (residual PET-CT uptake) following two cycles of ABVD. The majority of patients (6/9) achieved CR with a negative PET scan at the completion of treatment, whereas three patients exhibited disease refractoriness and began salvage chemotherapy. Eight patients (11.6%) relapsed by the end of this study, 3 patients (4.3%) had refractory disease, 3 patients (4.3%) had an early relapse, and 2 patients (2.9%) had a late recurrence. Six patients were administered salvage chemotherapy ifosfamide, carboplatin, and etoposide (ICE), whereas two patients were administered vinorelbine and gemcitabine. Three patients achieved remission after the second line of therapy, while five patients got the third line: three received DHAP (dexamethasone, cytarabine, and Platinol), one received ICE, and one patient received vinorelbine and gemcitabine. At 3 years, the OS and EFS were 91.9% and 83.6%, respectively as shown in Fig. 1. The OS and EFS for our investigated patients based on different prognostic factors are shown in Tables 2, 3 and Fig. 2.

**Table 2:** Overall survival and its relation to different prognostic factors

**Factors**		**OS %**				**Median (months)**	
	***n***	**6 m**	**1 yr**	**2 yrs**	**3 yrs**	**95% *CI***	***p*-value**
**All**	69	98.6	95.5	91.9	91.9	NA	NA
**Gender**							
Male	57	98.2	96.4	92.2	92.2	53.4 (31.4–75.5)	0.895
Female	12	100	90.9	90.9	90.9	NA	
**Age groups**							
0–5 yrs	13	100	92.3	92.3	92.3	NA	**0.038**
> 5–10 yrs	29	96.6	92.8	92.8	92.8	55.2	
> 10–15 yrs	21	100	100	100	100	53.5	
≥ 15 yrs	6	100	100	40.0	40.0	23.4 (8.4–38.5)	
**ESR**							
< 30	44	97.7	92.8	92.8	92.8	55.2	0.524
≥ 30	25	100	100	90.4	90.4	53.4	
**LN no**							
< 3	40	100	100	100	100	NA	**0.008**
≥ 3	29	96.6	89.4	81.3	81.3	55.0	
**Extra nodal**							
No	37	100	100	100	100	NA	**0.017**
Yes	32	96.9	90.6	83.3	83.3	NA	
**Liver involvement**							
No	62	98.4	96.7	94.3	94.3	55.2	**0.008**
Yes	7	100	85.7	71.4	71.4	53.4	
**Bone marrow involvement**							
No	62	98.4	96.7	92.6	92.6	55.2	0.350
Yes	7	100	85.7	85.7	85.7	53.5	
**Lung involvement**							
No	63	100	98.3	96.7	96.7	55.2	** < 0.001**
Yes	6	83.3	66.7	33.3	NA	23.4 (6.5–40.2)	
**Bulky disease**							
No	56	98.2	98.2	93.6	93.6	55.2	0.245
Yes	13	100	84.6	84.6	84.6	NA	
**Stage**							
I–II	33	100	100	100	100	NA	**0.032**
III–IV	36	97.2	91.5	85.0	85.0	NA	
**B symptoms**							
No	45	100	97.6	97.6	97.6	55.2	**0.033**
Yes	24	95.8	91.7	81.7	81.7	NA	
**CMT**							
No	47	97.9	93.3	89.9	89.9	NA	0.720
Yes	22	100	100	95.5	95.5	53.4 (24.1–82.7)	
**Histopathology subtypes**							
MC	29	100	96.4	96.4	96.4	NA	0.258
NS	36	97.2	94.3	87.5	87.5	55.2	
**Radiotherapy dose**							
15	15	100	93.3	93.3	93.3	53.4	0.495
20	7	100	100	100	100	NA	
**Interim PET-CT**							
Negative	60	100	96.5	94.7	94.7	55.2	0.068
Positive	9	88.9	88.9	88.9	74.1	NA	
**Interim negative & CMT**							
No	43	100	95	95	95	100–100	0.35
Yes	17	100	100	100	100	88.4–100	

*CMT* combined modality treatment, *MC* mixed cellularity, *NS* nodular sclerosis, *ESR* erythrocyte sedimentation rate, *LN* lymph node, *PET-CT* positron emission tomography-computed tomography

**Table 3:** Event-free survival and its relation to different prognostic factors

Factors		EFS%				Median (months)	*p*-value
	*n*	6 m	1 yr	2 yrs	3 yrs	(95% CI)	
**All**	69	94.0	90.7	88.8	83.6	NA	NA
**Gender**							
**Male**	57	96.4	92.5	90.3	83.8	NA	0.413
**Female**	12	81.8	81.8	81.8	81.8	NA	
**Age groups**							
**0–5 yrs**	13	92.3	92.3	92.3	92.3	NA	0.178
**> 5–10 yrs**	29	96.4	92.4	92.4	92.4	NA	
**> 10–15 yrs**	21	95.2	95.2	89.9	80.0	NA	
**≥ 15 yrs**	6	80	53.3	53.3	53.3	NA	
**ESR**							
**< 30**	44	92.9	90.3	87.4	87.4	NA	0.997
**≥ 30**	25	96.0	90.4	90.4	76.2	NA	
**LN no**							
**< 3**	40	100	100	96.9	96.9	NA	0.005
**≥ 3**	29	85.2	76.4	76.4	68.8	NA	
**Extra nodal**							
**No**	37	100	100	96.6	96.6	NA	0.013
**Yes**	32	87.1	79.5	79.5	70.7	NA	
**Splenic involvement**							
**No**	44	97.6	97.6	94.8	94.8	NA	0.013
**Yes**	25	87.5	77.7	77.7	66.6	NA	
**Bone marrow involvement**							
**No**	62	93.3	89.7	87.5	87.5	NA	0.950
**Yes**	7	100	100	100	80.0	NA	
**Lung involvement**							
**No**	63	93.5	91.8	89.9	84.6	NA	0.371
Yes	6	100	66.7	NA	NA	NA	
**Bulky disease**							
**No**	56	94.4	90.4	88.1	80.1	NA	0.595
**Yes**	13	92.3	92.3	92.3	92.3	NA	
**Stage**							
**I–II**	33	100	96.2	96.2	96.2	NA	0.029
**III–IV**	36	88.2	81.4	81.4	73.3	NA	
**B symptoms**							
**No**	45	100	97.6	94.7	86.1	NA	0.059
**Yes**	24	82.6	77.1	77.1	77.1	NA	
**CMT**							
**No**	47	93.3	90.7	87.6	87.6	NA	0.909
**Yes**	22	95.5	90.9	90.9	83.3	NA	
**Histopathology subtypes**							
**MC**	29	100	100	100	100	NA	0.005
**NS**	36	88.2	81.7	78.0	66.8	NA	
**Radiotherapy dose**							
**15**	15	93.3	86.7	86.7	74.3	NA	0.196
**20**	7	100	100	100	100	NA	
**Interim PET-CT**							
**No**	60	93.2	91.4	91.4	84.8	NA	0.351
**Yes**	9	100	87.5	74.1	74.1	NA	
**Interim negative & CMT**							
**No**	43	100	90	90	90	80.7–99.2	0.719
**Yes**	17	94	94	88.2	88.2	72.9–100	

*CMT* combined modality treatment, *MC* mixed cellularity, *NS* nodular sclerosis, *ESR* erythrocyte sedimentation rate, *LN* lymph node, *PET-CT* positron emission tomography-computed tomography

## Discussion

This study included 69 patients whose ages at diagnosis ranged from 3 to 17 years, with a median age of 8 years; 60% of our patients were below the age of 10 years (18.8% were younger than 5 years, and 42.1% were between 5 and 10 years). There are considerable differences in the clinical and pathological features of pediatric HL patients based on geographic region. The average age of diagnosis in the Western world is between 12 and 15 years [5–7]. Patients in developing countries tend to present at a younger age. Other studies find comparable ages of diagnosis in developing nations in Africa and Asia, demonstrating a link between HL and infection with the Epstein-Barr virus in these regions [7–9].

There were 82.6% male and 17.4% female, resulting in a male-to-female ratio of 4.7:1. Numerous publications [10, 11] have documented male predominance in HL, as did our study, which was comparable to a Pakistan study that revealed a male-to-female ratio of 3.6:1 [8]. The cause for this male dominance should be explored thoroughly. A possible explanation is that gender discrimination is still common in many developing countries. Nodular sclerosis histology was the major pathologic subtype (47.8%), followed by mixed cellularity histology (24.1%), in contrast to other developing countries where mixed cellularity is the prevalent subtype, as described by Faizan et al. [8]. Patients with mixed cellularity and nodular sclerosis had an EFS of 100% and 66%, respectively, with *P* = 0.005. This was comparable to a previous research in which patients with mixed cellularity histology had a 4-year EFS rate of 95.2%, which was considerably higher than that of patients with nodular sclerosis histology (75.7%; *P* = 0.008). However, there was no statistical significance regarding OS in our sample [12]. With a threshold of 10 g/dl, the hemoglobin level of all patients was measured. Anemia was detected in 39 individuals (56.5%), which is slightly higher than the 23.1% rate reported by Mondello et al. This may be attributed to the low socioeconomic status and poor nutrition of a significant number of our patients [13]. At diagnosis, 47.8% of our cohort’s patients were in stages I and II, whereas 52.2% were in stages III and IV. Our population has a greater rate of advanced stages compared to others [14]. The difference in our analysis was a more advanced state at presentation, mostly as a result of a delay in diagnosis and referral to pediatric oncologists [5, 8, 15, 16]. In contrast, the majority of newly diagnosed patients in the Western world are in the early stages of disease (stages I–II) [11]. The goal of new therapeutic approaches for pediatric patients with HL is to significantly enhance cure rates while minimizing treatment-related early and late adverse effects. Initial therapeutic approaches for children contain high doses of radiation. In the GPOH-HD 95 research, radiation was only administered to patients who did not achieve complete remission following chemotherapy [17]. Current treatment strategies are based on risk- and response-adjusted therapy, with patients receiving chemotherapy with or without low-dose radiation therapy. Between 1975 and 2010, the 5-year survival rate for pediatric HL improved from 81 to  > 95% [18]. However, in underdeveloped countries, survival is still much lower due to late presentation, inadequate supportive care, and the absence of accessible targeted treatment as salvage therapy, those whose conventional chemotherapy has failed die of disease progression [7, 19].

There are few published studies from Egypt; thus, we undertook the present study to describe outcomes in the Egyptian community, particularly for those treated with response-adapted therapy. Until the end of 2016, all patients in our cohort received combined modality; thereafter, response-based treatment was adopted, and radiation therapy was only administered to patients with SER. The majority of patients in our research were RER patients who attained full remission with PET-CT negative following the second chemotherapy cycle (60 patients). Patients with RER had superior 3-year OS and EFS compared to those with SER (94.7% vs. 74.1%) and (84.8% vs. 74.1%); *P* = 0.068 and *P* = 0.351, respectively). These results were consistent with those of Gallamini et al., who reported that the 3-year EFS for RER vs. SER was 95% vs. 82%, respectively, and with those of another study, in which the 3-year EFS was 73.6% vs. 64%, indicating that a positive interim PET has a significant impact on survival [20, 21].

In the present study, CMT (chemotherapy plus radiation) was administered to 22 patients (31.9%), whereas chemotherapy alone was administered to 47 patients (68.1%). The OS and EFS of patients who got CMT were marginally greater than those who did not (95.5% vs. 89.9%) and (87.6% vs. 83.3%), respectively, although these differences were not statistically significant (*P* = 0.720 and 0.909). This was comparable to a research conducted by Metzger et al. on 86 patients, in which patients who did not undergo irradiation were estimated to have a 5-year EFS of 89.4%, which was comparable to those who did undergo irradiation (87.5%) [22], and in agreement with another randomized research conducted by Jhawar et al., who revealed that 5-year OS was 97.3% for patients receiving CMT and 94.5% for those getting chemotherapy alone (*P* = 0.001) [23]. In contrast, Ali et al. [9] stated that radiation is the single independent predictor for inferior OS, as determined by multivariate analysis. The 5-year OS and EFS rates for those who had received radiation treatment were 93.4% and 80%, respectively, compared to 30% and 30% for patients who got chemotherapy alone (*P* < 0.0001) [9, 24]. We found that of the sixty patients who obtained RER despite a negative interim PET, seventeen received combined modality therapy, since CMT was the standard of care regardless of response at that time. The 3-year OS and EFS rates of patients who achieved RER and got CMT were 100% and 88.2%, respectively, compared to 95% and 90% in patients who achieved RER but only received chemotherapy (*P* = 0.35 for OS and *P* = 0.71 for EFS). Therefore, radiation can be omitted from RER without compromising treatment outcomes. In the present study, radiation was administered to 22 patients (31.9%), with 15 patients (68.2%) receiving a dosage of 15 Gy and 7 patients (31.8%) receiving a dose of 20 Gy or more. The 3-year OS and EFS of patients who got 15 Gy were (93.3% and 74.3%), respectively, but patients who received 20 Gy or more had a 3-year OS and EFS of 100% and 100%, respectively; however, the *P*-values were not statistically significant (*P* = 0.495 & 0.196%). In a different research, the 5-year EFS for patients treated with low-dose radiation treatment (15–25 Gy) was 84%, whereas the 5-year EFS for patients treated with standard dosage (25–35 Gy) was 81%. Consequently, it appears that the outcomes of standard-dose and low-dose radiation are equal; thus, minimizing the dosage to reduce the risk of toxicity should be addressed [25]. Thus, we may apply low-dose radiation without affecting the result while also reducing toxicity. The indication for radiation differs for patients with bulky mediastinal mass; in the COG study AHOD0831, participants with SER or initial bulky areas received radiation, but in the EuroNet-PHL trial, only patients with SER received radiotherapy. In our cohort, there were eight patients with mediastinal enlargement; all of them obtained full remission on interim PET-CT. Three patients had a combined modality, and none experienced relapse; five patients did not get radiation, and only one experienced relapse and attained CR following salvage chemotherapy. Although our sample size was small, we can support the omission of radiotherapy for those with bulky mediastinal mass and achieved RER to avoid late toxicity in the form of cardiotoxicity, pulmonary toxicity, and secondary malignancy, particularly in adolescent females with a higher incidence of breast and thyroid cancer; however, a larger sample size is still required to support our results [26]. As shown in Table 4, the OS and EFS at 3 years for the entire cohort were 91.9% and 83.6%, which is comparable to international studies and more favorable to other developing nations. In the present study, four patients died, with neutropenic sepsis being the cause of death in two patients and illness progression being the cause of death in the other two; effective supportive care is one of the most significant factors in preventing treatment-related mortality.

**Table 4:** Comparison of the current study with other pediatric HL studies conducted in low-middle-income countries

Author	Country	Protocol	Radiotherapy	EFS (%)	OS (%)
Present study	Egypt	ABVD	No Rth for RES	90	95
			15 Gy for SER	74.1	88.9
Tariq Ghafoor et al. (2018) [27]	Pakistan	OEPA/COPDAC	19.8 Gy	82.4	91.2
			10 Gy boost		
Faizan et al. (2016) [8]	Pakistan	OEPA/COPDAC	19.8 Gy	84	92
			10 Gy boost		
Fadoo et al. (2010) [15]	Pakistan	COPP/ABVD	Does not mentioned	94	94
Faizan et al. (2016) [8]	UK	OEPA/COPDAC	19.8 Gy	92	100
			10 Gy boost		
Arya et al. (2006) [16]	India	COPP/ABVD	20–25 Gy	87.9	91.5
Trehan et al. (2013) [5]	India	MOPP, COPP, and ABVD	Dose not mentioned	77.7	92.7
Bhethanabhotla et al. (2017) [10]	India	ABVD	25 Gy to bulky site	84.8	95.3
Sherief et al. (2015) [11]	Egypt	ABVD	21–35 Gy	84.7	96.6
Geel et al. (2017) [28]	South Africa	COPP/ABVD	14–44 Gy		79
		OEPA/OPPA			
		CHIVPP			
Castellanos et al. (2014) [6]	Central America	ABV/COPP	No	71	
Zubizarreta et al. (2017) [29]	Argentina	ABVD	25 Gy	84	95
Samy Elbadawy et al. (2008) [30]	Egypt	OPPA, E-OPA/ COPP	20–30 Gy	86.1	95.3
Amany M. Ali et al. 2018 [24]	Egypt	ABVD/COEP	15 Gy for CR	71.8	81.8
			25.5 Gy for PR		

*ABVD* Adriamycin, bleomycin, vinblastine, dacarbazine. *OEPA* vincristine, etoposide, prednisone, doxorubicin (Adriamycin). *COPDAC* cyclophosphamid, vincristine (Oncovin), dacarbazine, prednisone. *COPP* cyclophosphamid, vincristine (Oncovin), procarbazine, prednisone. *MOPP*, mechlorethamine, vincristine, procarbazine, and prednisone. *OPPA* vincristine, procarbazine, prednisone, doxorubicin (Adriamycin). *CHIVPP* chlorambucil, vinblastine, procarbazine, prednisolone. *ABV* Adriamycin, bleomycin, vinblastine. *COEP* cyclophosphamid, vincristine (Oncovin), etoposide, prednisone

## Conclusions

HL is an extremely curable disease; the population in developing countries presents more in younger age groups and at advanced stages. We have comparable 3-year OS and EFS rates that approach the international results. The omission of radiotherapy in response-adaptive strategy can be safely done without compromising treatment results. Patients with bulky mediastinal mass can be treated according to a response-adapted approach without affecting the outcome, but a larger sample size and a longer period of follow-up are required to confirm this issue. Finally, it is important to prioritize HL management in low-income countries. All attempts to enhance diagnostic and treatment results are substantial and highly desired in the setting of a highly curable disease.

## Data Availability

The datasets used for the current study are available from the corresponding author on reasonable request.

## Abbreviations

ABVD: Adriamycin, bleomycin, vinblastine, and dacarbazine
ABV: Adriamycin, bleomycin, and vinblastine
CHIVPP: Chlorambucil, vinblastine, procarbazine, and prednisone
CMT: Combined modality treatment
COEP: Cyclophosphamid, Oncovin, etoposide, and prednisone
COPDAC: Cyclophosphamid, Oncovin, dacarbazine, and prednisone
COPP: Cyclophosphamid, Oncovin, procarbazine, and prednisone
CR: Complete response
CT: Computed tomography
CTH: Chemotherapy
CXR: Chest x-ray
DHAP: Dexamethasone, high-dose ara-C, and Platinol
EFS: Event-free survival
ESR: Erythrocyte sedimentation rate
HL: Hodgkin lymphoma
HR: High risk
ICE: Ifosfamide, carboplatin, and etoposide
IR: Intermediate risk
ISRT: Involved site radiotherapy
LN: Lymph node
LR: Low risk
MC: Mixed cellularity
MOPP: Mechlorethamine, vincristine, procarbazine, and prednisone
NCI: National Cancer Institute
NS: Nodular sclerosis
OEPA: Vincristine, etoposide, prednisone, and Adriamycin
OPPA: Vincristine, procarbazine, prednisone, and Adriamycin
OS: Overall survival
PET: Positron emission tomography
RER: Rapid early responder
RTH: Radiotherapy
SER: Slow early responder
